# A Novel Polyester Varnish Nanocomposites for Electrical Machines with Improved Thermal and Dielectric Properties Using Functionalized TiO_2_ Nanoparticles

**DOI:** 10.3390/ma16196478

**Published:** 2023-09-29

**Authors:** Hanaa M. Ahmed, Nagat M. K. Abdel-Gawad, Waleed A. Afifi, Diaa-Eldin A. Mansour, Matti Lehtonen, Mohamed M. F. Darwish

**Affiliations:** 1 Basic Science Department, Faculty of Engineering at Shoubra, Benha University, Cairo 11629, Egypt; hana.ahmed@feng.bu.edu.eg; 2 Department of Electrical Engineering, Faculty of Engineering at Shoubra, Benha University, Cairo 11629, Egypt; prof.nagat@gmail.com (N.M.K.A.-G.); enr_waleed@yahoo.com (W.A.A.); 3 Egyptian Railways Maintenance and Services Company, Egyptian National Railways, Cairo 11678, Egypt; 4 Department of Electrical Power Engineering, Egypt-Japan University of Science and Technology (E-JUST), Alexandria 21934, Egypt; 5 Department of Electrical Power and Machines Engineering, Faculty of Engineering, Tanta University, Tanta 31511, Egypt; 6 Department of Electrical Engineering and Automation, School of Electrical Engineering, Aalto University, 02150 Espoo, Finland

**Keywords:** breakdown strength, dielectric and thermal properties, electrical insulation, functionalized and un-functionalized nanoparticles, nanocomposites, polyester varnish

## Abstract

Recently, there has been a growing interest in polymer insulating materials that incorporate nanoscale inorganic additives, as they have shown significantly improved dielectric, thermal, and mechanical properties, making them highly suitable for application in high-voltage insulating materials for electrical machines. This study aims to improve the dielectric and thermal properties of a commercial polyester varnish by incorporating different concentrations of titanium dioxide nanoparticles (TiO_2_) with proper surface functionalization. Permafil 9637 dipping varnish is the varnish used for this investigation, and vinyl silane is the coupling agent used in the surface functionalization of TiO_2_ nanoparticles. First, nanoparticles are characterized through Fourier transform infrared spectroscopy to validate the success of their surface functionalization. Then, varnish nanocomposites are characterized through field emission scanning electron microscopy to validate the dispersion and morphology of nanoparticles within the varnish matrix. Following characterization, varnish nanocomposites are evaluated for thermal and dielectric properties. Regarding thermal properties, the thermal conductivity of the prepared nanocomposites is assessed. Regarding dielectric properties, both permittivity and dielectric losses are evaluated over a wide frequency range, starting from 20 Hz up to 2 MHz. Moreover, the AC breakdown voltage is measured for varnish nanocomposites, and the obtained data are incorporated into a finite element method to obtain the dielectric breakdown strength. Finally, the physical mechanisms behind the obtained results are discussed, considering the role of nanoparticle loading and surface functionalization.

## 1. Introduction

Polymers have become key enabling materials for multiple applications because of their dependability, availability, ease of manufacture, and low cost. One of these promising applications is electrically insulating materials [1,2,3,4]. In the field of electrical machines, either motors or generators, polymers are used in the form of varnish or resin as embedded insulation. Vacuum-impregnated polyester varnish, in particular, is a type of organic electrical insulating material that helps strengthen the insulating system’s mechanical integrity, enhance its dielectric and thermal conductivity, and protect its internal coils and components from outside influences such as moisture, pollution, and other impacts [5]. 

There are several desired properties in polymer insulating materials, such as high dielectric strength, low dielectric loss, high thermal conductivity, high mechanical strength, oxidation resistance, etc. In order to improve these properties, polymer insulating materials can be modified with various additives and curing agents [6,7]. Due to their tiny size and surface impact, inorganic nanoparticles can be used to improve these properties [8,9]. Highly dispersing tiny amounts of nanoparticles throughout a polymeric matrix form what is called polymer nanocomposites, with characteristics and performance determined by the type of nanoparticles, their size, surface treatment, and concentration. Furthermore, it has been determined that the interaction area located at the interface between the base material matrix and the fillers is crucial in determining the characteristics of the synthesized nanocomposites [10,11]. Metal oxides represent the majority of types of nanoparticles used to improve the characteristics of polymer nanocomposites, such as titania (TiO_2_), zinc oxide (ZnO), alumina (Al_2_O_3_), etc.

Because of its stability and electrical properties, titanium dioxide (TiO_2_) is now extensively used as a filler in polymer nanocomposites [12]. It manifests as a white powder with a density of 4.26 g/cm^3^ and a particle molecular weight of 79.87 g/mol. It possesses high thermal conductivity as well as a large dielectric constant [13]. Incorporating TiO_2_ nanoparticles into organic varnish may encounter various obstacles, including the challenge of achieving a homogeneous dispersion of the nanoparticles within the polymeric matrix. This difficulty arises from the nanoparticles’ large surface area, which can cause agglomeration clusters to form, leading to inadequate mechanical and electrical characteristics of the composite material [14]. The most popular method for reducing nanoparticles’ surface energies and increasing their compatibility with the polymer matrix is surface functionalization, which ensures uniform nanoparticle dispersion and stabilization within the base matrix. This can be achieved using a suitable coupling agent with a surface tension close to the nanoparticle and polymer matrix. For most organic polymers as a matrix and TiO_2_ as metal oxide nanoparticles, vinyl silane, and amino silane meet this requirement, and they were selected for nanoparticle surface functionalization in previously reported studies [15,16,17,18,19].

Numerous studies have explored the mechanical, thermal, and dielectric characteristics of solid and liquid nanocomposite insulators for application in high-voltage insulating materials for electrical machines. However, there has been a relatively low amount of research aiming to improve the properties of impregnating resin nanocomposites and insulating varnishes.

Edison and Pugazhendhi [20] demonstrated that incorporating a small weight percentage of zirconia (ZrO_2_) nanoparticles in an organic varnish improved the permittivity, dielectric strength, and thermal properties of the examined varnish.

Prabath and Sameera [21] have demonstrated that the addition of small amounts of silica nano-fillers can enhance the dielectric strength of silica-epoxy resin nanocomposites, with the nanocomposite containing 2.0 wt.% of silica (SiO_2_) nanoparticles exhibiting the highest breakdown strength. However, increasing the nanoparticle concentration beyond 5.0 wt.% significantly lowers the dielectric breakdown strength. A similar study was conducted by Lei and Song [22] on silica-containing epoxy resin, and it revealed that doping 0.5 wt.% SiO_2_ nanoparticles improved the AC dielectric strength and dielectric losses of the polymeric material. Nevertheless, a higher doping ratio led to a region of nanoparticle surfaces overlapping, causing a reduction in the epoxy’s dielectric performance. Bazrgari and Tayebi [23] also demonstrated that nanoparticle insertion impacts the characteristics of the epoxy matrix, since introducing 1.0 vol.% of Al_2_O_3_ nanoparticles into the epoxy matrix enhanced the flexural strength, stiffness, and impact strength of the nanocomposite. In addition, the epoxy matrix’s wear rate and coefficient of friction are considerably lowered as a result of the addition of Al_2_O_3_ nanoparticles. However, upon adding 3.0 vol.% to the epoxy matrix, these advantages were not seen due to the nanoparticle’s aggregation taking place at this concentration.

The previously mentioned studies and others reported in the literature did not deeply discuss the improvement of polyester varnishes upon adding TiO_2_ nanoparticles as one of the more common modern nanoparticles, heavily incorporating electrical insulating base materials, and the effect of nanoparticle surface functionalization on thermal, dielectric, and breakdown strength characteristics of synthesized nanocomposites.

This paper presents an evaluation and analysis of the most significant thermal and dielectric parameters of nanocomposites synthesized through the incorporation of various doping ratios of functionalized and unfunctionalized TiO_2_ nanoparticles into vacuum-impregnated polyester varnish. The quantities of interest include thermal conductivity, dielectric permittivity (ὲ), dissipation factor (tan δ), AC breakdown strength (AC-BDS), and Weibull statistical analysis for breakdown voltage. These properties are compared with those of the neat varnish to assess the effectiveness of the obtained nanocomposites.

The novelty of this paper can be summarized as follows:

(1) Surface functionalization of TiO_2_ nanoparticles using a vinyl silane coupling agent and their subsequent incorporation into the selected polyester varnish matrix.
(2) Fabrication of varnish/TiO_2_ nanocomposite samples with several loadings of functionalized and un-functionalized TiO_2_ varying from 0% to 0.7% (*w*/*w*).
(3) A novel approach aimed to enhance the thermal and electrical properties of a polyester varnish that is employed in the electric machine’s insulation winding.
(4) Key properties of interest include thermal conductivity, dielectric permittivity, dissipation factor, AC-BDS, and Weibull analysis of breakdown voltage.

Furthermore, a comparative analysis is performed against the neat insulating material to assess the effectiveness of the developed nanocomposites. Thus, this work will contribute significant insights into the impact of nanoparticle incorporation and functionalization on the performance enhancement of the insulating varnishes, highlighting the potential for advanced insulation materials in electric machines. The findings presented herein offer valuable prospects for further advancements in the field of electrical insulation, making this study highly valuable for researchers and practitioners in the domain. 

## 2. Experimental Section

The process of nanocomposite preparation involves optimizing the type and weight fraction of nanoparticles to achieve the most significant improvement in the desired characteristics. Therefore, it is crucial to select an appropriate preparation technique to obtain accurate results and draw precise conclusions from the evaluation of the nanocomposites.

### 2.1. Materials

In the current investigation, Permafil 9637 dipping with vacuum-impregnated thermoset polyester has been used as the base varnish. It is a solvent-free electrical insulating varnish obtained from the Von Roll group, New York City, NY, USA. This varnish is commonly used for treating transformers, stators, fields, armatures, and coils. As per the manufacturer data sheet, the viscosity of this insulating material is 250 cP, its flash point is 302 K, its density is 0.970 g/cc, its average solids are 48%, its processing temperature is 423 K, and its maximum service temperature in the air is 453 K [24]. The solvent used for the selected varnish (the thinner) was mineral spirits (Rule 66 type), which are a mixture of aliphatic hydrocarbons with less than 1% aromatics. Regarding the TiO_2_ nanoparticles, they were selected for this study based on two main factors. Firstly, TiO_2_ nanoparticles possess a high permittivity, which endows them with a remarkable ability to create deep traps for charge trapping. This characteristic significantly enhances the dielectric strength of the material. The enhanced dielectric strength of polymer nanocomposites filled with TiO_2_ nanoparticles was confirmed in [25] compared to those filled with magnesium oxide (MgO), ZnO, and Al_2_O_3_ nanoparticles. Secondly, TiO_2_ nanoparticles are known for their facile surface functionalization process, allowing functionalized TiO_2_ nanoparticles to be used frequently in dielectric nanocomposites [26,27]. In addition, the authors tested SiO_2_ nanoparticles with varnish in a previous study [28], and it exhibited a maximum enhancement in the dielectric strength of about 12.6%. TiO_2_ nanopowder (size = 21 nm and 99.5% purity) was purchased from Sigma Aldrich, St. Louis, MO, USA. Further, tris (2-methoxy ethoxy) vinyl silane is used as a coupling agent in the surface functionalization stage of nanoparticles, and methane sulfonic acid is used in the surface activation of nanoparticles before their reaction with the coupling agent. Both were purchased from Merck group, Burlington, MA, USA. Further, iso-propanol alcohol, toluene, and 20% butyl acetate as solvents were purchased from different suppliers. No specific treatments had been carried out for the used chemicals and reagents.

### 2.2. Nanoparticles Functionalization

Surface modification of nanoparticles via coupling agents is a common approach to improving the interfacial interaction between the nanoparticles and the base material. In this particular investigation, vinyl silane was employed to modify the surface of TiO_2_ nanoparticles [29,30]. Two sequential preparatory stages are used to conduct this surface modification: the initial stage is the activation of the TiO_2_ nanoparticle’s surface with hydroxyl groups by applying an acid etching technique, which is followed by modifying these activated nanoparticles with vinyl silane. The activation process involves mixing 5 g of TiO_2_ with 50 mL of 10% methane sulfonic acid and heating the mixture for four hours at 110 °C, then spinning a Hitachi centrifuge under 1200 rpm for five minutes to extract the powdered “activated nanoparticles”. The collected powder was then washed multiple times using deionized water and then dried overnight at 120 °C in a vacuum oven.

**Figure 1:**
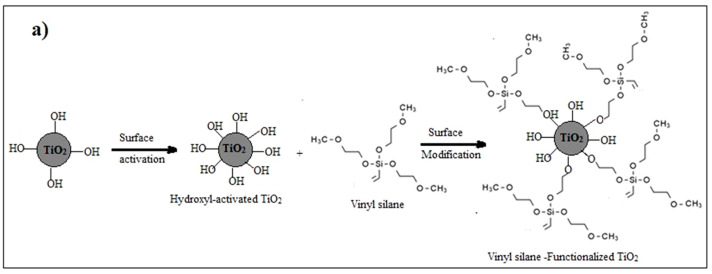

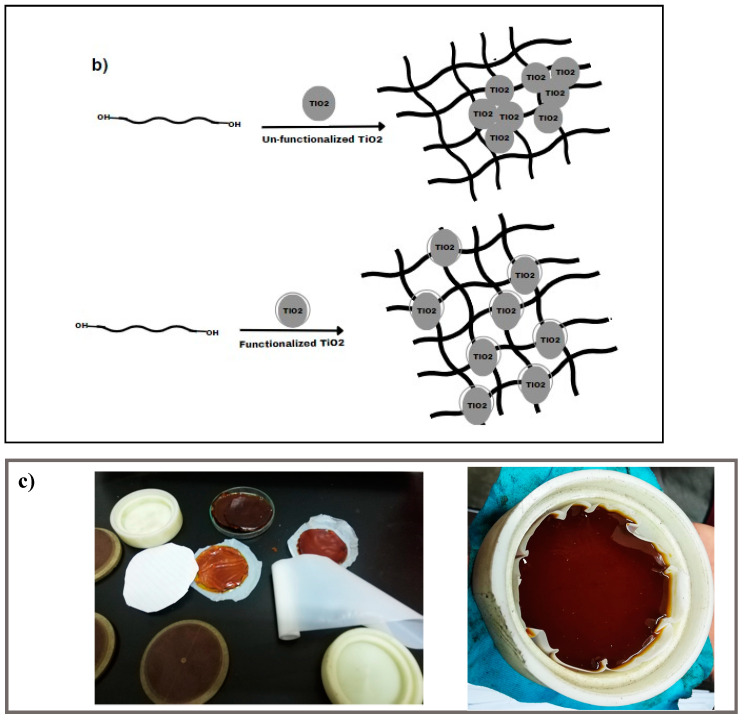
(**a**) Functionalization process of TiO_2_ nanoparticles by vinyl-silane; (**b**) Synthesis of TiO_2_-varnish nanocomposites; and (**c**) Laboratory preparation samples.

In the second step, 1.5 g of the previously activated nanoparticles were sonicated in 30 mL of toluene in an Elmasonic sonicator water bath for 30 min, and the mixture’s temperature was maintained at 70 °C during the sonication. The solution was then subjected to mechanical stirring for 2 h at 70 °C and 500 rpm. Then, a 10% weight fraction vinyl silane solution in toluene was gradually added, and the mixture was stirred for 8 h at the same temperature. For further characterization and utilization of the functionalized TiO_2_, it was collected, cleaned with isopropyl alcohol, and then dried at 120 °C for 24 h in a vacuum oven, as illustrated in Figure 1a.

### 2.3. Nanocomposites Preparation

Varnish/TiO_2_ nanocomposites were synthesized through the solution-casting method, a simple and flexible approach that is utilized to obtain nanocomposite materials on a small scale [31]. In this technique, a determined amount of polyester varnish was dissolved well in a suitable amount of the chosen volatile solvent (thinner).

In another vessel, a suitable amount of TiO_2_ nanopowder (functionalized or un-functionalized) was mixed in a suitable amount of a thinner and sonicated at 30 °C for 30 min. By the end of sonication time, the suspended nanoparticles were added to the previously prepared varnish/thinner mixture, and the entire mixture was vigorously stirred. To prevent air bubbles and voids, the mixture was defoamed for a few seconds at a speed of 1200 rpm after being violently mixed for 5 min at a speed of 1500 rpm in the planetary centrifuge mixer. The combination was then subjected to 75 min of sonication in an Elmasonic sonicator at around 50 °C to promote optimal dispersion of the nanoparticles within the varnish and to enhance the homogeneity of the obtained nanocomposites. The resulting mixture was then cast into a non-stick Teflon dish to avoid adhesion and kept at room temperature for 2 h before being annealed under vacuum at 130 °C for 8 h to accelerate the solvent evaporation. As the used varnish is a thermoset polymer type, the samples get stronger after curing due to the devolving of chemical linkages created between their components, as seen in Figure 1b.

In this study, seven different varnish/TiO_2_ nanocomposites were obtained with nanoparticle concentrations ranging from 0% to 0.7%. (*w*/*w*) (nanoparticles/varnish) using functionalized and un-functionalized nanoparticles, as shown in Figure 1b. Further, Figure 1c presents photographs of the prepared samples inside the laboratory. It is important to note that the authors exerted significant efforts to ensure small variations in thickness among different samples, where the diameter is constant for all samples that equal 8 cm and the sample thickness varies within the range of 1.5–2 mm.

### 2.4. Samples Characterization and Measurement

The chemical structure of the un-functionalized and functionalized TiO_2_ nanoparticles was studied using the Fourier Transform Infrared (FT-IR) technique with BRUKER ALPHA PLATINUM ATR Infrared Spectroscopy at wave number ranges of 400–4000 cm^−1^. FT-IR Spectroscopy is a nondestructive optical technique used mostly for specific tasks, including determining a molecule’s fundamental characteristics, analyzing known species quantitatively within a substance, identifying compounds, and elucidating structures. This optical method is based on measuring the amount of Infrared (IR) radiation transmitted or absorbed by a certain material, which is mainly because of the vibrational and rotational bands of absorption of its molecules as a function of wavelength (λ) or its reciprocal, which is commonly known as wavenumber (ν). 

The crystallography of the vinyl-functionalized TiO_2_ nanoparticles compared to that of the un-functionalized TiO_2_ nanoparticles was studied using X-ray diffraction (XRD). The XRD spectra of both nanopowdered samples were obtained using an X-ray diffractometer (X’Pert PRO, PANalytical, Almelo, The Netherlands) with Cu K_α_ radiation in the angular range of 2θ = 4° to 80°. The X-ray diffractometer was operated at 40 kV with a 0.02° angular step. In the obtained diffractogram, the peak positions were analyzed and compared to known crystallographic databases (card Nos. 96-900-8215 and 96-900-8215) to determine the crystal structure of the tested samples.

On the other hand, the dispersion degree of nanoparticles within the varnish was examined by a device called QUANTA FEG 250 Field Emission Scanning Electron Microscope, abbreviated by FE-SEM.

Regarding thermal properties, thermal conductivity was measured based on the transient plane source (TPS) method using the Hot Disk TPS 500 S instrument. This technique provides accurate measurement due to its ability to remove contact resistance’s effect. The measurements were accomplished at various temperatures: 293 K, 313 K, 333 K, and 353 K. Where thermal conductivity measurement is not influenced by variations in sample thickness.

The dielectric properties of nanocomposites were evaluated in comparison to neat varnish using an Agilent E4980A precision LCR meter at a wide frequency range of 20 Hz to 2.0 MHz. The equivalent parallel resistance (R_p_) and capacitance (C_p_) are the electrical parameters that were measured for all prepared samples, and then the most crucial dielectric characteristic parameters, which are dissipation factor (tan δ), dielectric loss (ε″), and permittivity (ὲ), can be calculated according to:

ε′ = d × C_p_/(A × ε_o_)

and

ε″ = d/(2π f ε_o_ A R_p_)

where (C) means the capacitance of the capacitor filled with the dielectric material, (f) represents the frequency, and (ε_o_) is the permittivity of free space (=8.85 × 10^−12^ F/m). This equation takes into account the thickness of the sample (d) and the cross-sectional area of the electrodes (A), which are important parameters that affect the capacitance. Therefore, ε′ and ε″ can be further used to calculate the values of the relative permittivity (ε_r_) and dissipation factor (tan δ) of the dielectric material according to the Havriliak-Negami equation: ε_r_ = ε′ − iε″ and tan δ = ε″/ε′ [32,33].

The necessary AC-BDS was evaluated by two methods for comparison and to guarantee accurate calculations, i.e., a measurement method using the general formula of E = V/d and a simulation method using a finite element technique that modeled the quazi-uniform electric field, obtained using a mushroom-to-mushroom electrode shape. Using this electrode shape, the electrostatic field can be approximated as a uniform field, thereby being calculated using the formula E = V/d, where V depicts the root-mean-square value of the necessary observed breakdown voltage (BDV) and d is the sample thickness. The electrode system, including the sample, was immersed in a dielectric fluid to avoid a possible flashover across the sample surface. The test was performed per ASTM-D149-09 [34]. The finite element approach was used to obtain the AC-BDS from the measured BDV [35,36,37].

## 3. Characterization of Nanoparticles and Varnish Nanocomposites

The polyester used in this study is a hydroxyl functional unsaturated polyester with a general chemical structure of HO-(R-O-CO-CH=CH-CO-O-)n-R-OH, where “n” represents the number of repeating units in the polymer and “R” represents the alkyl or aryl groups of the diol or polyol of the polyester. It ended with hydroxyl (-OH) groups on both sides of its structure, and these hydroxyl groups help in the curing of polyester by reacting with each other or with other additives. Moreover, hydroxyl groups in the polymer chain enable the formation of hydrogen bonds between polymer chains and any additives or between polymer chains themselves. This can form cross-linked products and thermoset polymers. These cross-linked products can have improved properties, such as higher strength, toughness, and chemical resistance.

The synthesis of a nanocomposite material using TiO_2_ nanoparticles and hydroxyl-functional unsaturated polyester can be achieved through various methods. One common method is the solution blending technique, which involves dissolving the hydroxyl-functional unsaturated polyester in a suitable solvent and adding the TiO_2_ nanoparticles to the solution. The mixture is then stirred for a period of time to ensure homogeneity and allow the nanoparticles to interact with the polymer chains. The solution is then cast onto a substrate and allowed to dry or cure at elevated temperatures to form a solid nanocomposite material. During the mixing process, the hydroxyl groups in the polymer chains can react with the surface hydroxyl groups on the TiO_2_ nanoparticles through H-bonding and chemical bonding. This can lead to the formation of a strong interface between the nanoparticles and the polymer matrix, resulting in improved thermal, mechanical, and barrier properties of the nanocomposite material.

In the case of using vinyl silane-functionalized TiO_2_ nanoparticles in the preparation of TiO_2_/varnish nanocomposite, the interaction between TiO_2_ nanoparticles and the hydroxyl polyester gets stronger due to the presence of chemical reactions between the unreacted methoxy ethoxy groups on the nanoparticles and the hydroxyl groups on the polyester. This chemical interaction strengthens the interfacial zone between the nanoparticles and the polymer matrix, which in turn improves the dispersion of nanoparticles, with a positive impact on various properties of nanocomposites, as will be seen in the following sections.

### 3.1. FT-IR Spectroscopy

**Figure 2:**
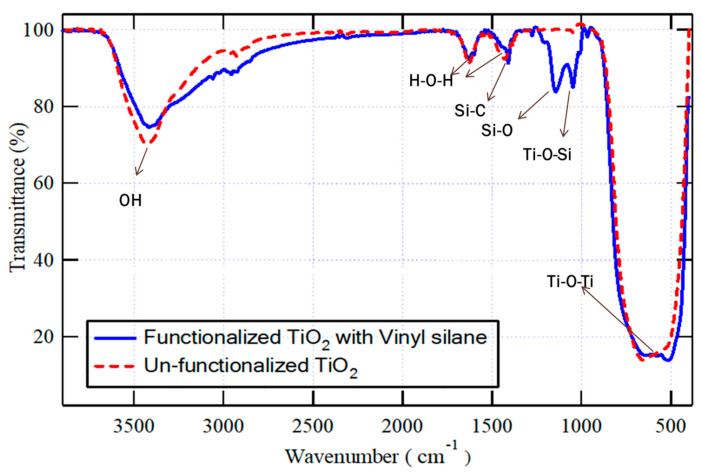
FT-IR for both vinyl-functionalized and un-functionalized TiO_2_ nanoparticles in the selected wavenumber ranges.

Both vinyl-functionalized and un-functionalized TiO*_2_* nanoparticles were analyzed using FT-IR, as demonstrated in Figure 2. In both samples, the peak observed at wavenumber 3500 cm^−1^ corresponds to the stretching vibrations of O-H, and the weak peaks appear at 1630 cm^−1^ and 1430 cm^−1^ correspond to the scissoring vibration of adsorbed water (H-O-H), which are interfered respectively with the stretching vibration of C=C and the scissoring vibration of =CH_2_ bonds; however, the absorption band at 700 cm^−1^ corresponds to the vibration of Ti-O-Ti bonds. However, in the spectra of the functionalized TiO*_2_* nanoparticles, two additional absorption peaks were detected at around 1300 cm^−1^ and 1000 cm^−1^, which correspond to Si-O and Ti-O-Si bonds. These peaks indicated that the vinyl silane coupling agent could successfully interact with the surface of nanoparticles. The unreacted methoxy ethoxy groups in vinyl silane, Si-C, and Si-O bonds, appear respectively at the same absorption bands of H—O-H (1430 cm^−1^) or Si-O of the silanol group of functionalized TiO_2_ (1300 cm^−1^) [38].

### 3.2. XRD of TiO_2_ Nanoparticles

**Figure 3:**
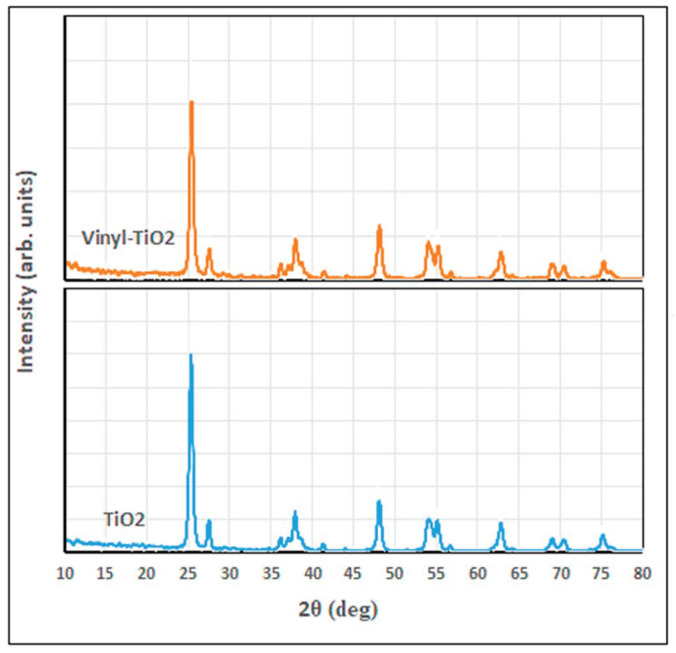
XRD diffractograms of TiO_2_ and the functionalized Vinyl TiO_2_ nanoparticles.

Figure 3 shows the XRD diffractogram of unfunctionalized and functionalized TiO_2_. As shown in this figure, both tested samples show a well-known reflection pattern for the anatase crystalline phase: (101), (004), (200), (105), (211), (204), (116), (220), (215), located respectively at 2θ of 25.3, 37.6, 47.8, 53.7, 54.8, 62.6, 68.7, 70.2, and 75.0°, while the small peak appeared at 27.1° characteristic for the rutile (110) phase [7,38,39]. These peaks confirm the presence of the corresponding crystalline phases of TiO_2_ in the sample; however, the modification of TiO_2_ nanoparticles has not significantly altered the crystallography of the vinyl-silane modified-TiO_2_ structure except for the appearance of the peaks located at 2θ of 41.2 and 56.5° for vinyl-TiO_2_.

### 3.3. Nanocomposites Morphology

The surface morphology of the synthesized nanocomposite samples was characterized using a device called field emission scanning electron microscopy, abbreviated by FE-SEM, using the Quanta FEG-250 model (FEI-Inc., Billings, MT, USA). It has a wide field of view and is provided with an S150A SPUTTER COATER for sample preparation [40]. Further, for the sake of comparison between un-functionalized and functionalized TiO_2_, we have adopted the lowest concentration (varnish/0.1% TiO_2_ nanocomposites) for two reasons. Firstly, at low concentrations, individual nanoparticles are more dispersed within the polymer varnish matrix, allowing for a more detailed examination of their distribution and dispersion, thereby providing valuable insights into the effectiveness of nanoparticle functionalization and the interactions between the nanoparticles and the polymer varnish matrix. Secondly, capturing SEM images at low concentrations shifts the focus of agglomeration and dispersion behavior towards the nanoparticle’s structure and morphology rather than being dependent on concentration. This distinction is crucial in order to assess the impact of nanoparticle functionalization accurately and mitigate the confounding effect of concentration on agglomeration and dispersion behavior.

**Figure 4:**
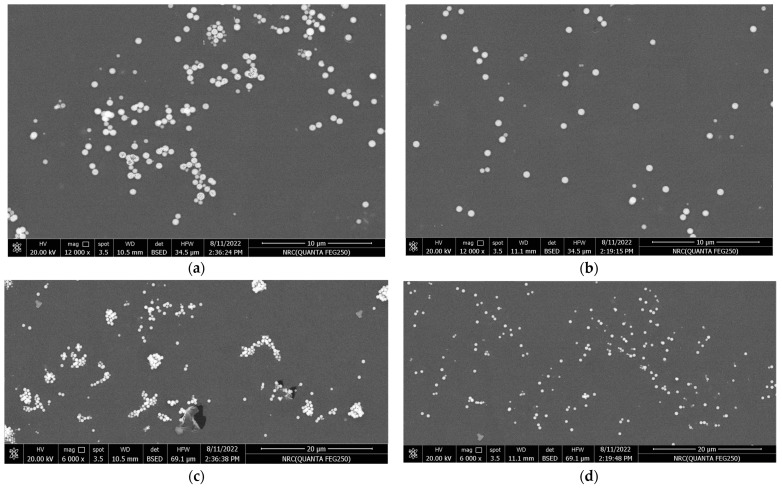
FE-SEM micrographs of Varnish/TiO_2_ nanocomposites: (**a**,**c**) are for 0.1% un-functionalized TiO_2_ at magnifications 12,000× and 6000×, respectively, and (**b**,**d**) are for 0.1% functionalized TiO_2_ at 12,000× and 6000×, respectively.

In Figure 4, polished cross-sections of TiO_2_-containing polyester nanocomposites with 0.1 wt.% of un-functionalized and functionalized nanoparticles were scanned by FE-SEM. In addition, various magnifications of 6000× and 12,000× are selected for accurate examination of TiO_2_ dispersion within the polymer varnish matrix (see Figure 4a–d). Functionalized TiO_2_ nanoparticles were better dispersed in the base material compared to the un-functionalized ones. Additionally, some TiO_2_ nanoparticle aggregates were observed within the varnish matrix for the un-functionalized nanoparticles, suggesting that the compatibility between varnish chains and nanoparticles has been enhanced. This improved compatibility can alter the morphological structure of the varnish nanocomposites and enhance their properties, which will be discussed later.

The reason for this improvement may be attributed to the similarity in surface tension between the vinyl silane grafted on the surface of TiO_2_ nanoparticles and the base varnish material, which enhances the hydrophilic character of the organic-based varnish.

## 4. Thermal Properties

The generation of heat in electrical equipment results from various types of losses, including those from moving components (friction), magnetic losses that occur in the core, polarization, and conduction losses in the insulation, and resistive losses in the winding and insulation. Due to smaller designs and higher power densities, thermal management in electrical equipment has become increasingly challenging. Inadequate thermal design, leading to elevated temperatures, can reduce the load-bearing capabilities and service life of the equipment.

Therefore, comprehending the fundamental thermal properties of materials is critical to material design and research. The three primary parameters for assessing thermal properties in electrical insulation are thermal expansion, thermal conductivity, and thermal endurance [41].

When selecting an insulating material used in the insulation of electrical components, thermal conductivity is an important key since it is essential in dissipating heat generated by electrical components. Thus, it is advantageous to utilize dielectric materials with increased thermal conductivity to minimize overheating at winding hotspots and to avoid loss of dielectric characteristics under thermal stress. The addition of nanoparticles to insulating varnish can significantly alter its thermal conductivity depending on the size, shape, type, and concentration of the nanoparticles, as well as the nature of the polymer matrix [42,43]. In general, the thermal conductivity of the resulting nanocomposites increases above that of base polymers due to the high thermal conductivity of nanoparticles. This effect is more pronounced at higher nanoparticle concentrations, where the nanoparticles form a continuous network within the polymer matrix, allowing efficient heat transfer. However, the thermal conductivity enhancement may also depend on the nature of the interface between the polymer matrix and the nanoparticles. A strong interface can promote efficient heat transfer, while a weak interface can hinder it. Therefore, surface functionalization of nanoparticles will further enhance the thermal conductivity of nanocomposites.

By including the appropriate nano or microfillers, their performance may be modified [44,45]. In contrast to micro-scale composites, the substantial specific surface area of nano-fillers might result in a significant contribution to interfacial heat resistance in a nanocomposite. The behavior of thermal conductivity in polymer nanocomposites can be complex, and it depends on several factors, such as the size and shape of the nanoparticles and their dispersion within the polymer matrix [46,47]. In this section, we will discuss in more detail the effect of nanoparticle surface modification and their concentrations on the thermal conductivity of polyester varnish as an insulating material, which was the focal point of this investigation.

**Figure 5:**
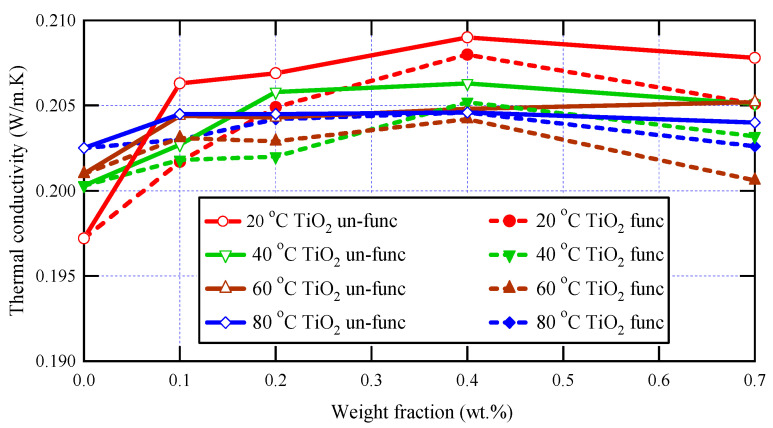
Thermal conductivity analysis for varnish nanocomposites and neat varnish.

The thermal characteristics of the prepared samples were studied using the transient plane source technique, which is a robust and powerful technique for studying materials’ thermal conductivity. The measurements were implemented at various temperatures: 293 K, 313 K, 333 K, and 353 K, and the obtained thermal conductivity values for varnish nanocomposites containing functionalized and un-functionalized TiO_2_ are presented in Figure 5 at various nanoparticle loadings (0, 0.1, 0.2, 0.4, and 0.7 wt.%).

Thermal conductivity is not only affected by temperature but also by other factors, such as the material’s chemical structure, the crystal’s purity, and so on [44]. Due to the complicated topology of polymer chains, bulk polymers typically have very low thermal conductivities. Blending polymers with highly heat-conductive nanoparticles is a popular technique for improving thermal conductivity [45]. 

It is clear in this figure that, for all tested samples, the thermal conductivity decreases as temperature increases, and this is because the thermal conductivity of most materials, including nanocomposites, is related to the rate of heat transfer by phonon transport, which is affected by temperature [48,49]. At low temperatures, phonons are the dominant carriers of heat in a material, and the material’s thermal conductivity tends to increase as the temperature increases. However, at higher temperatures, additional mechanisms can come into play, such as thermal vibrations, electron-phonon interactions, and boundary scattering, which can reduce the rate of heat transfer by phonons and decrease the thermal conductivity.

The obtained data shown in Figure 5 also demonstrate the dependence of thermal conductivity on the presence of nanoscale materials within the polymeric matrices of polyester varnish, and it is clear that, for all applied temperatures, all synthesized nanocomposites have greater thermal conductivity values than that of the neat polyester varnish sample because the nanoparticles act as effective heat conductors and enhance phonon transport through the composite material. Furthermore, due to the greater thermal conductivity of nanoparticles, the thermal conductivity rises at low concentrations and increases with increasing nanoparticle loading since the high concentration of well-dispersed nanoparticles can create a network of heat-conducting pathways that facilitate heat transfer and lead to an increase in thermal conductivity [50,51] This increase was limited up to 0.4 wt.%, after which the thermal conductivity values started to decrease due to the scattering of phonons at the interfaces between the nanoparticles and the polymer matrix, which can lead to a reduction in thermal conductivity, particularly at high temperatures where phonon scattering is more prevalent [52]. The highest thermal conductivity value was observed for the nanocomposite sample containing 0.4 wt.% of un-functionalized TiO_2_, with a notable enhancement reaching 6% compared to the neat varnish.

Regarding the effect of the functionalization of nanoparticles on the thermal conductivity of nanocomposites, it is observed in Figure 5 that the thermal conductivities of nanocomposites containing un-functionalized nanoparticles have greater values than those in the case of functionalized nanoparticles, and this was clear at all temperatures and for all nanoparticle loadings. These observations may be attributed to the great interfacial surfaces of functionalized TiO_2_ nanoparticles, which create higher levels of phonon scattering where heat propagates extremely slowly, which in turn decreases the thermal conductivity of the tested nanocomposites. Furthermore, the higher values of thermal conductivity in the case of un-functionalized nanocomposites over the functionalized ones can be attributed to a greater number of agglomerates, which act as rapid heat conduction zones. This tendency is influenced by the aggregation of fillers and the filler-matrix interfacial thermal resistance.

To conclude, the incorporation of functionalized TiO_2_ nanoparticles can potentially enhance the thermal conductivity of the varnish, leading to better heat dissipation and improved performance of the system. Moreover, thermal conductivity may also display a non-monotonic rising trend as a function of filler concentration [4,5,6,7]. One possible reason for a non-monotonic rising trend is the presence of filler-filler interactions that can affect the thermal conductivity of the composite material. At low filler concentrations, the thermal conductivity may increase as the concentration of filler particles increases due to the increased number of heat-conducting pathways created by the filler particles. However, as the concentration of filler particles increases further, the interactions between the filler particles may begin to have a negative effect on the thermal conductivity, leading to a decrease or leveling off of the thermal conductivity with increasing filler concentration. Another possible reason for a non-monotonic trend is the presence of filler-matrix interactions that can affect the thermal conductivity of the composite material. At low filler concentrations, the filler particles may be well-dispersed within the matrix material, leading to an increase in thermal conductivity as the concentration of filler particles increases. However, at higher concentrations, the filler particles may begin to agglomerate or cluster together, leading to a decrease in thermal conductivity due to the formation of regions with poor heat conductivity.

## 5. Dielectric Properties

### 5.1. Permittivity and Dielectric Loss

Significant enhancements in the dielectric properties of nanocomposites are desirable for replacing conventional insulation systems. Improvements in the dielectric properties of nanocomposites may result from a variety of causes, including changes in the local structure at interfaces between nanoparticles and polymer matrix, as well as changes in the density and depth of charge trapping sites, which have an impact on the stability of the trapped state and charge mobility. There may also be an increase in the likelihood that scattering mechanisms will occur. The benefits of wide interfacial areas in nanocomposites are mostly dependent on addressing new obstacles to achieving homogenous dispersion of nanoparticles while preventing particle aggregation. The dielectric constants of nanocomposites obtained at room temperature employing functionalized TiO_2_ nanoparticles were compared with those obtained using un-functionalized TiO_2_ nanoparticles.

The link between the electric flux density and the electric field intensity is described by dielectric permittivity, which is a constant proportionality. Permittivity becomes a complex quantity when a time-varying electric field is applied to a dielectric substance. The amount of energy the external field has stored in the material is indicated by the real component of the relative permittivity (also identified as the dielectric constant). In contrast, the imaginary relative permittivity describes the dielectric losses connected to the polarization and orientation of electrical dipoles. The dissipation factor, also known as the dissipation factor (tan δ), is the ratio between the imaginary and real components of the relative permittivity. Power equipment should employ dielectric materials with strong dielectric strength, a low value of the dielectric constant, and low dielectric losses or dissipation factors. To maintain an appropriate level of homogeneity in the electric field distribution, especially in heterogeneous systems, the choice of the dielectric constant must be carefully considered.

The introduction of nanofillers or any other modifications to the dielectric material can significantly affect the behavior of its permittivity with respect to frequency. This is because the molecular structure of the dielectric material is responsible for the polarization phenomena [53,54].

Dielectric parameters ε′ and tan δ impact the dielectric response of specific materials; therefore, in this investigation, the dielectric parameters for all synthesized samples were determined at ambient temperature and in a frequency range of up to two megahertz.

**Figure 6:**
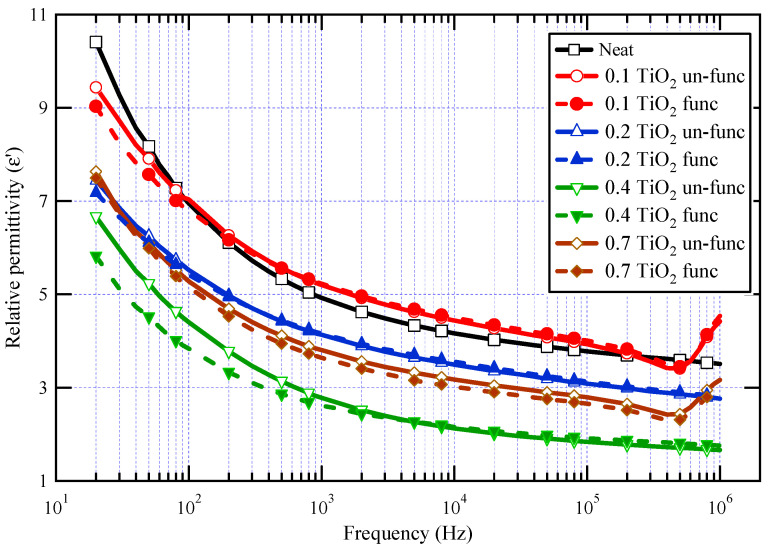
ε′ for neat varnish and varnish/TiO_2_ nanocomposites for different loading of nanoparticles.

The real component of the complex permittivity (ε′) for neat varnish as well as varnish/TiO_2_ nanocomposites samples is demonstrated in Figure 6. For all samples, it is demonstrated that as frequency increases, ε′ decreases. This behavior is expected for dielectric materials because the contribution of orientation polarization decreases as frequency increases [16,17,18,19,55,56]. The real value of dielectric permittivity is proportional to the number of oriented dipoles present in the tested material and their capacity to undergo orientation at the applied frequency. When the frequency rises, there will not be sufficient time for dipoles to follow the applied field and contribute to the orientation polarization, resulting in a decrease in ε′ rates [57,58,59]. However, the irregular increase in relative permittivity values in the megahertz range is attributed to the possible resonance effects resulting from insufficient contact in the measuring cell [60,61].

The results also demonstrated that the relative permittivity for almost all synthesized nanocomposites is lower than that of the neat varnish material, which is triggered by inhibiting the resin molecules owing to the presence of nanoparticles [62]. It can also be shown that, when the doping filler percent of TiO_2_ grows, the real permittivity of the prepared nanocomposites declines first and subsequently increases. The lowest permittivity was obtained at a nanofiller loading of 0.4 wt.%. The increase in permittivity at 0.7 wt.% TiO_2_ may be attributed to a decrease in the interface area contact between varnish and TiO_2_ nanofillers caused by nanoparticle agglomeration [63]. These agglomerations facilitate mobility and result in further molecular polarization.

The polarization of the base material is affected by two mechanisms: the first mechanism represents the limiting of chain mobility in the region of the nanoparticles through a few nanometers around the filler surface, and the second mechanism is the influence of the filler’s relative permittivity. The first process, which explains why relative permittivity decreases, is caused by the interface polymer layer as well as altered molecular structure and chain dynamics as compared to the base material polymer matrix. As a result, dipolar groups have no power in the presence of nanoparticles [64]. The first mechanism is relevant up to 0.4 wt.%, whereas the second mechanism takes over at higher concentrations.

It is significant to note that varnish/functionalized nanoparticles have lower ε′ values than varnish/un-functionalized nanoparticles for the same nanoparticle loading percent. This demonstrates that TiO_2_ has been chemically functionalized, which aids in their dispersion, improves their compatibility with the polymeric matrix, and ultimately limits chain mobility when exposed to an electric field. The same figure also displayed the impact of TiO_2_ nanoparticle concentration on varnish’s dielectric behavior. It is obvious that the relative permittivity values of nanocomposites vary with the TiO_2_ content, particularly in the case of nanocomposites containing functionalized TiO_2_ and in the low-frequency zone. It is further demonstrated that nanocomposites containing 0.4% TiO_2_ have the lowest ε′ values, particularly when functionalized TiO_2_ is present, which exhibits a reduction in ε′ of roughly 42% from neat varnish at 50 Hz.

**Figure 7:**
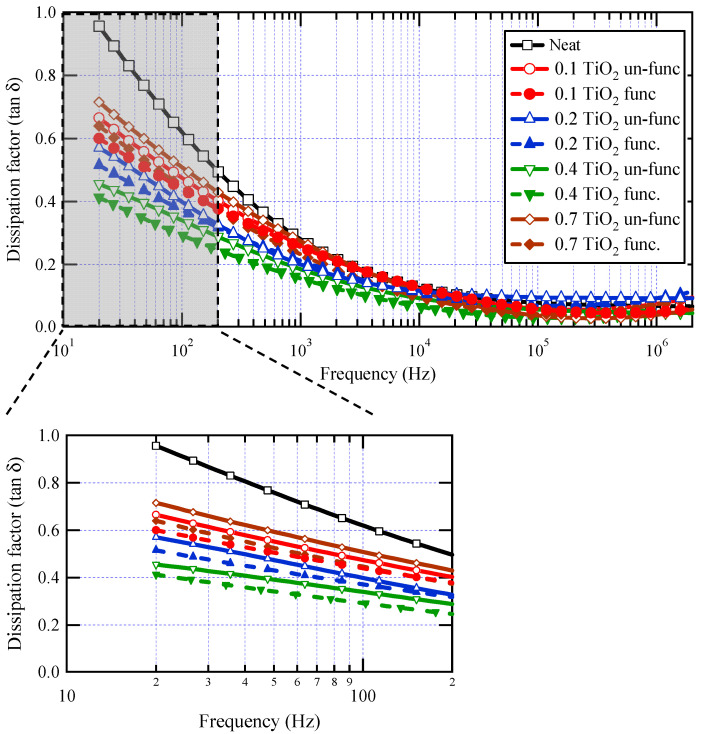
Dissipation factor for neat varnish as well as varnish/TiO_2_ nanocomposites for various filler contents.

Figure 7 represents the dissipation factor (tan δ) as a function of frequency at different weight fractions with and without surface functionalization. It is clear that there is a drop in tan δ values against frequency for all samples, likely due to a reduction in electrical conductivity in varnish nanocomposites with increasing frequency, which is brought on by charge carriers’ inability to pass through the thickness of the material at higher frequencies. There is an obvious reduction in tan δ values for all weight fractions when compared to base varnish material, especially at lower frequencies. With increasing the loading of TiO_2_ nanofillers in the base material, tan δ reduced up to 0.4 wt.% filler concentration, above which tan δ began to increase again at 0.7 wt.% filler concentration. For all weight fractions of nanoparticles, it is evident that the tan δ values of functionalized TiO_2_ nanoparticles are lower than those of un-functionalized ones.

These findings are attributed to the development of several interface areas in the composite by doping a tiny quantity of TiO_2_ nanoparticles. These interface areas reduce relative permittivity by restricting polarization in their proximity. Interface areas, on the other hand, operate as charge carrier trapping centers, reducing dielectric loss. However, with increasing nanofiller loading, an overlap occurs between interface zones, resulting in a loss of the beneficial effects of nanoparticles. With surface functionalization of nanoparticles, the dispersion of nanoparticles becomes better, which enriches the interface areas with a positive impact on the measured properties. The distinction is more visible at low frequencies. Table 1 depicts the percentage reduction obtained for tan δ values at a frequency of 50 Hz. The highest decrease in dielectric losses was about 56%, observed at 0.4 wt.% of functionalized nanoparticles. The limited impact of nanoparticles at high frequencies is a common observation in most previously published works on polymer nanocomposites [16,18,56,62].

### 5.2. AC Breakdown Strength

Mushroom-mushroom test cells were used to obtain dielectric breakdown strength tests. The test followed the procedures specified in the ASTM (D149-09) standard [34]. A total of ten tests were carried out for each sample, and then the average values were calculated. The synthetic sample’s AC breakdown voltage (AC-BDV) was measured directly using a uniform field of mushroom-mushroom electrodes and then divided by the sample thickness to obtain the AC-BDS. After that, another method is implemented to simulate the AC-BDS using the numerical finite element approach (FEM), and then the AC-BDS from measured and simulated methods are compared.

The electrostatic field is computed using the formula E = V/d for mushroom-mushroom arrangement as well as the approximation that the predicted sample thickness d means exceedingly thin with relation to the diameter of the electrode and V is the r.m.s value of the measured BDV. Moreover, Equation (1) may be used to calculate the observed breakdown strength values more precisely [28]:

(1) $$\frac{E_{1}}{E_{2}}={\left(\frac{d_{1}}{d_{2}}\right)}^{-n}$$

where *E*_1_ and *E*_2_ are the electric fields at the obtained thicknesses d_1_ and d_2_, respectively, and n is an exponent, which is assumed to be 0.7 based on the results in [65].

For a better breakdown of voltages in statistical summary, the Weibull distribution analysis is conducted for each tested sample, which is extended 10 times for one sample. The cumulative Weibull probability function for each sample, *F*(*v*), can be obtained from Equation (2). where *v* represents the breakdown voltage in kV, *ξ* denotes the shape parameter, and *λ* means the scale parameter in kV [33].

(2) $$F\left(v\right)=1-e^{-(\frac{v}{\lambda }{)}^{\;\xi }}$$

**Figure 8:**
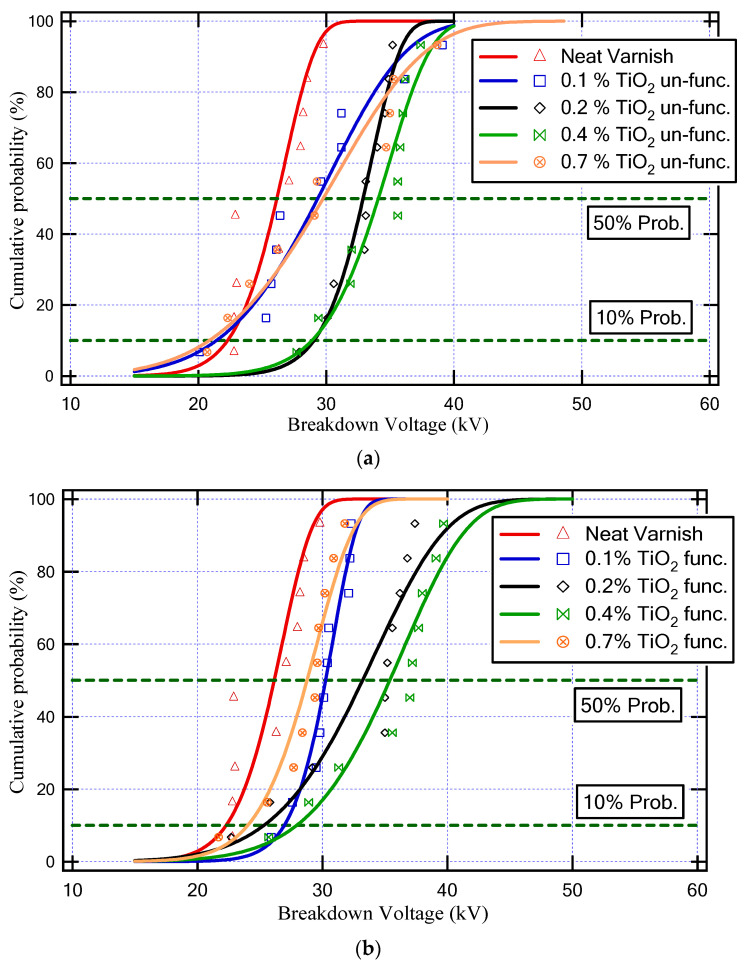
Weibull cumulative probability against breakdown voltage for various neat varnish and varnish/TiO_2_ nanocomposite samples; (**a**) unfunctionalized TiO_2_ and (**b**) functionalized TiO_2_.

Figure 8 demonstrates the Weibull cumulative probability versus breakdown voltage for various neat varnish and varnish/TiO_2_ nanocomposite samples. Furthermore, Table 2 depicts the values of BDV with AC supply at 50% probability and BDV at 10% probability for all varnish samples. Where the BDV values at 50% probability indicate the average value of BDV, and the minimum possible breakdown voltage happens at 10% probability of the BDV value. 

As a result, the varnish sample that has a higher BDV is (Varnish + 0.4% TiO_2_ (func.)), as underlined in Table 2. This sample achieved an enhancement in the BDV at a 50% probability of about 35% and at a 10% probability of about 25% compared to the neat varnish.

**Figure 9:**
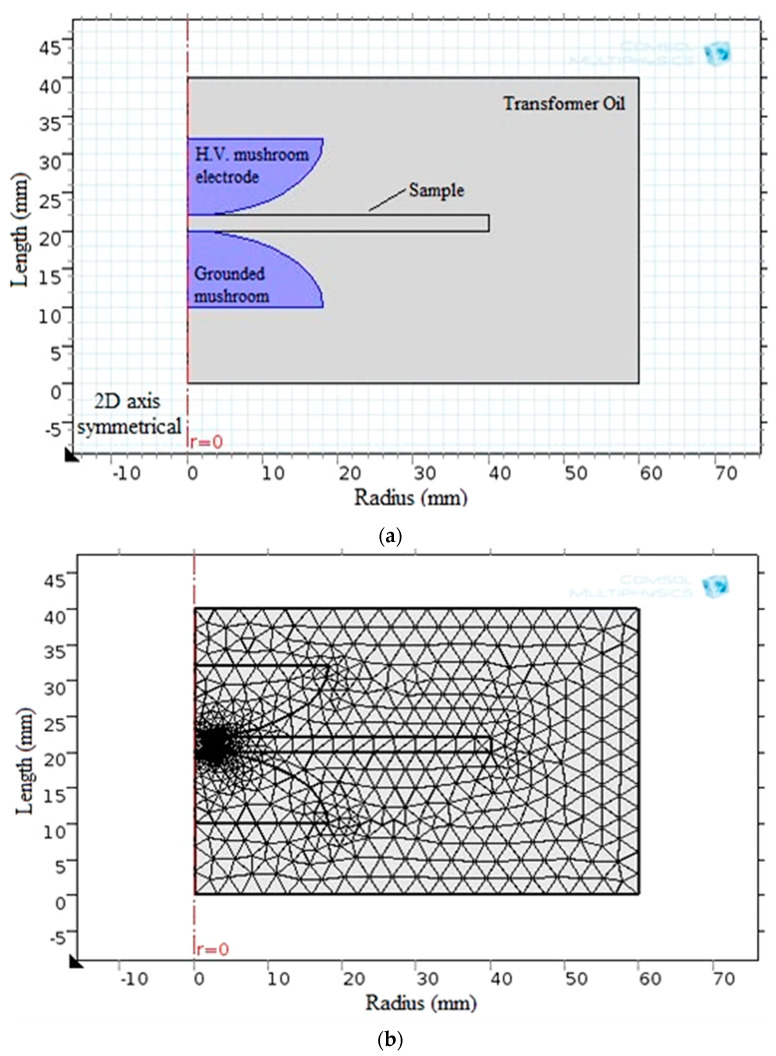
BDS computation via (**a**) the obtained model and (**b**) the finite element meshes shape.

Regarding the simulation analysis of breakdown strength, COMSOL Multiphysics 5.1 was used to successfully model the electrostatic field. Figure 9 depicts the model configuration and the corresponding generated finite element meshes.

The key input parameters for the model include the physical dimensions of the sample and electrodes and the sample’s relative permittivity (ε). In addition, the measured breakdown voltage is applied to the high-voltage electrode while the ground electrode is earthed. Then, FEM is used to obtain the AC-BDS. To apply FEM, the field region is first divided into smaller triangle parts, called meshes, to reduce the energy throughout the field region of interest. Equations (3)–(7) provide the electrical energy W stored within the full volume U of the region under study [28,62].

(3) $$w=\frac{1}{2}\int_{U}^{0}\varepsilon {\left|grad(V)\right|}^{2}.dU$$

(4) $$w=\frac{1}{2}{∭}_{U}^{0}\left[\varepsilon_{r}{\left[\frac{1}{r}.\frac{\partial rV_{r}}{\partial r}\right]}^{2}+\varepsilon_{\varphi }{\left[\frac{1}{r}.\frac{\partial V_{\varphi }}{\partial \varphi }\right]}^{2}+\varepsilon_{z}{\left[\frac{1}{r}.\frac{\partial {rV}_{z}}{\partial z}\right]}^{2}\right].drd\varphi dz$$

(5) $$\frac{w}{\varphi }=\frac{1}{2}.\varepsilon \iint_{A}^{0}\left[{\left[\frac{1}{r}.\frac{\partial rV_{r}}{\partial r}\right]}^{2}+{\left[\frac{1}{r}.\frac{\partial {rV}_{z}}{\partial z}\right]}^{2}\right]drdz$$

(6) $$\frac{w}{\varphi }=\frac{1}{2}.\varepsilon \sum_{i=1}^{n}\left[{\left[\frac{1}{r}.\frac{\partial rV_{r}}{\partial r}\right]}^{2}+{\left[\frac{1}{r}.\frac{\partial {rV}_{z}}{\partial z}\right]}^{2}\right]dA_{i}$$

(7) $$\vec{E}=-\nabla V=-\vec{I}\frac{\partial V(r,z)}{\partial r}-\vec{k}\frac{\partial V(r,z)}{\partial z}$$

where (∇^2^ V = 0) denotes the Laplacian equation, ((w)/(φ)) denotes the energy density per-elementary area dA, A_i_ is the area of the ith triangle element, and n denotes the total number of elements. As a result, the formulation for energy reduction across the whole system may be written as (∂(w⁄φ))/(∂(V(r,z)) = 0). Then, the electrostatic potential is obtained at the unknown potential nodes.

Table 3 displays the measured AC-BDS using the formula E = V/d and the generated AC-BDS from FEM at the mushroom electrode tip. The AC-BDS of neat varnish was 13.52 kV/mm, obtained from FEM, and increased for all varnish/TiO_2_ nanocomposite samples. The explanation for this is the significant interfacial area that exists between the polymeric matrix as well as the nanoparticles. Note that this varnish material is not the main insulation layer for machine conductors. Moreover, it is used to enforce machine winding insulation and mechanical rigidity.

**Figure 10:**
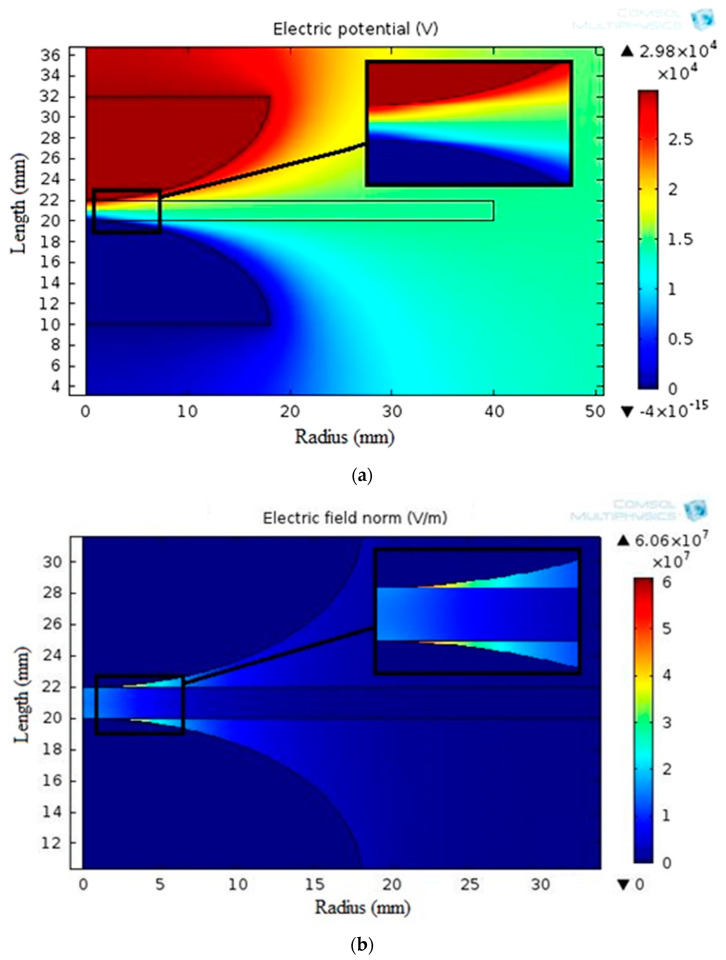
Distributions of (**a**) potential and (**b**) electrostatic field around mushroom-to-mushroom electrode for the varnish/0.4 wt.% TiO_2_ nanocomposite.

**Figure 11:**
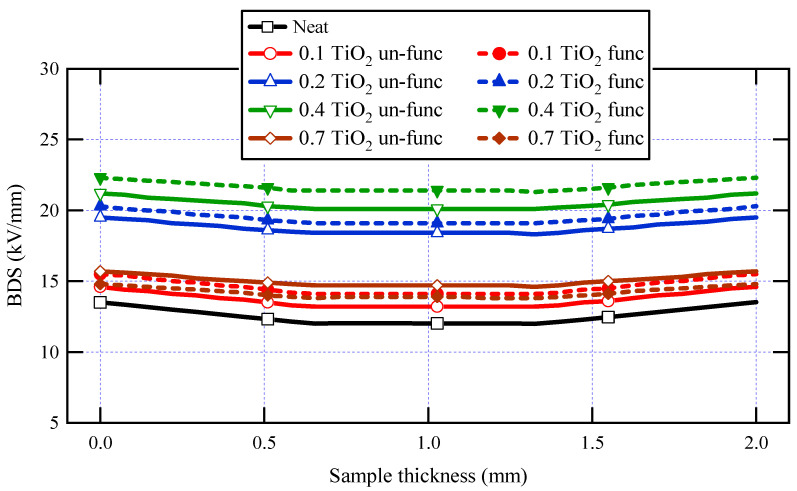
AC-Breakdown strength for neat varnish as well as varnish/TiO_2_ nanocomposites for various filler contents.

Figure 10 displays the simulated electric potential and electrostatic field distribution along the synthetic sample thickness for the varnish/TiO_2_ nanocomposite containing 0.4% TiO_2_ nanoparticles. Figure 11 presents the electrostatic field change along the thickness for all samples. According to the simulation results, the breakdown strength of all the nanocomposite samples created is greater than that of the basic varnish material.

It is significant to note that for almost all synthesized samples, varnish/functionalized nanoparticles have higher BDS values than varnish/un-functionalized nanoparticles when concentrating on the influence of functionalization on breakdown strength. This is due to the high interfacial area between the nanoparticles and the polymer matrix in functionalized samples and the excellent nanoparticle dispersion within the matrix. Another finding is that when the doping filler percent of TiO_2_ increases, the breakdown strength of nanocomposite samples increases first and subsequently decreases. The breakdown strength of the nanocomposite achieves its greatest value when the loading percent is 0.4 wt.% of functionalized nanoparticles. These findings are attributed to the interaction between base polymer chains and nanoparticles, resulting in a tiny interfacial layer around the nanoparticle. This layer is divided into two sections: a loose polymer area and a tightly bound region with restricted mobility [66]. 

With low filler loadings, a greater space exists between nanoparticles, and the loose polymer nanolayer facilitates the development of breakdown channels and movement of charge carriers between the electrodes, causing the breakdown strength to be lower than that obtained at 0.4 wt.%. On the other hand, with the greater number of nanofillers at 0.4 wt.%, the development of breakdown channels is hindered, and the movement of charge carriers is inhibited. The overlapping of the firmly bonded polymer regions in the interface as the inter-particle distances rise may be the reason for the breakdown strength decrease as nanoparticles’ loading increases [67].

On the other hand, when filler loading rises, the increasing heat conductivity of the nanocomposite may be the reason for the increase in AC-BDS. Greater heat dissipation due to enhanced thermal conductivity may result in increased breakdown strength.

## 6. Conclusions

The effect of integrating functionalized and un-functionalized TiO_2_ nanoparticles inside commercial varnish polymer matrices on both thermal and dielectric properties was investigated in the current study. Physical and chemical processing methods were used to fabricate varnish/TiO_2_ nanocomposites for electrical applications. The thermal and dielectric characteristics of varnish insulators have been enhanced by the addition of TiO_2_ nanoparticles up to a specified percentage of nanofillers. The functionalization of nanoparticles achieved more enhancements in dielectric properties and less in thermal ones.

Some significant conclusions are as follows:

(1) A considerable improvement in the thermal conductivity (K), real permittivity (ε′), dissipation factor (tan δ), and breakdown strength (AC-BDS) of synthesized nanocomposites was found based on the weight fraction of combined nanoparticles.
(2) In terms of AC-BDS, FEM is used to simulate the modeled non-uniform field with the configuration shape of a mushroom-mushroom electrode, which is then compared to the observed BDS values. It is found that the greatest percentage error between them does not exceed 2.88%.
(3) Surface functionalization of nanoparticles had a good impact on synthesized nanocomposites’ real permittivity (ε′), dissipation factor (tan δ), and AC-BDS but slightly decreased the thermal conductivity (K) property.
(4) Functionalized 0.4% wt. TiO_2_ is considered the best loading fraction for reducing polarization and losses within industrial insulating varnish, resulting in increased dielectric varnish breakdown strength of about 35.5% compared with neat varnish.
(5) Un-functionalized 0.4% wt. TiO_2_ is considered the best loading fraction for enhancing the thermal conductivity of the insulating polyester varnish, especially at low temperatures.

## Figures and Tables

**Table 1:** Percentage reduction obtained for tan δ values of varnish nanocomposites at a frequency of 50 Hz.

	0.1 wt.%	0.2 wt.%	0.4 wt.%	0.7 wt.%
Un-functionalized nanoparticles	27%	40%	53%	23%
Functionalized nanoparticles	36%	45%	56%	35%

**Table 2:** Weibull statistical analysis results for all varnish samples.

Varnish Samples	BDV at 50% (kV)	BDV at 10% (kV)
Neat Varnish	26.2	22.3
Varnish + 0.1% TiO_2_	29.4	21.5
Varnish + 0.1% TiO_2_ (func.)	30.3	27
Varnish + 0.2% TiO_2_	32.9	29.1
Varnish + 0.2% TiO_2_ (func.)	33.3	25.4
Varnish + 0.4% TiO_2_	34.1	28.9
Varnish + 0.4% TiO_2_ (func.)	35.5	28
Varnish + 0.7% TiO_2_	29.8	20.9
Varnish + 0.7% TiO_2_ (func.)	28.8	24.1

**Table 3:** AC-BDS of varnish nanocomposites and the neat varnish.

Sample Type	Measured B.D.V (kV)	Sample Thickness (mm)	ε_r_ at 50 Hz	Measured BDS (kV/mm)	Simulated BDS Using FEM (kV/mm)	Error * (%)
Neat Varnish	26.85	2.0	8.115	13.43	13.52	0.73
Varnish + 0.1% TiO_2_	28.36	2.0	7.911	14.18	14.61	2.94
Varnish + 0.1% TiO_2_ (func.)	30.42	2.0	7.57	15.21	15.38	1.1
Varnish + 0.2% TiO_2_	33.07	1.5	6.25	19.04	19.37	1.25
Varnish + 0.2% TiO_2_ (func.)	34.37	1.5	6.11	19.48	20.04	2.79
Varnish + 0.4% TiO_2_	34.48	1.5	5.23	20.69	21.17	2.26
Varnish + 0.4% TiO_2_ (func.)	36.12	1.5	5.02	21.68	22.25	2.56
Varnish + 0.7% TiO_2_	29.71	1.8	6.1	15.45	15.65	1.28
Varnish + 0.7% TiO_2_ (func.)	29.17	1.8	5.99	14.69	14.94	1.67

* Error is calculated as the percentage between measured and simulated BDS.

## Data Availability

The data presented in this study are available on request from the corresponding author.

## References

[1] Pleşa I., Noţingher P.V., Schlögl S., Sumereder C., Muhr M. (2016). Properties of Polymer Composites Used in High-Voltage Applications. Polymers.

[2] Li S.T., Yu S.H., Feng Y. (2016). Progress in and prospects for electrical insulating materials. High Volt..

[3] Wang J., Hu L., Li W., Ouyang Y., Bai L. (2022). Development and Perspectives of Thermal Conductive Polymer Composites. Nanomaterials.

[4] Sekula R., Immonen K., Metsä-Kortelainen S., Kuniewski M., Zydroń P., Kalpio T. (2023). Characteristics of 3D Printed Biopolymers for Applications in High-Voltage Electrical Insulation. Polymers.

[5] Calabrese E., Calabrese E., Raimondo M., Catauro M., Vertuccio L., Lamberti P., Raimo R., Guadagno L. (2023). Thermal and Electrical Characterization of Polyester Resins Suitable for Electric Motor Insulation. Polymers.

[6] Azizi S., Momen G., Ouellet-Plamondon C., David E. (2020). Performance improvement of EPDM and EPDM/Silicone rubber composites using modified fumed silica, titanium dioxide and graphene additives. Polym. Test..

[7] Gracia L., Beltran A., Errandonea D. (2009). Characterization of the TiSiO_4_ structure and its pressure-induced phase transformations: Density functional theory study. Phys. Rev. B.

[8] Zhou Y., Hu J., Chen X., Yu F., He J. (2016). Thermoplastic polypropylene/aluminum nitride nanocomposites with enhanced thermal conductivity and low dielectric loss. IEEE Trans. Dielectr. Electr. Insul..

[9] Ashokbabu A., Thomas P. Dielectric and thermal properties of PTFE/CaCu_3_Ti_4_O_12_(CCTO) nanocomposites. Proceedings of the IEEE 2019 International Conference on High Voltage Engineering and Technology (ICHVET).

[10] Idumah C.I., Obele C.M. (2021). Understanding interfacial influence on properties of polymer nanocomposites. Surf. Interfaces.

[11] Zhang W., Emamy H., Pazmiño Betancourt B.A., Vargas-Lara F., Starr F.W., Douglas J.F. (2019). The interfacial zone in thin polymer films and around nanoparticles in polymer nanocomposites. J. Chem. Phys..

[12] Rasool K., Rasool K., Usman M., Ahmad M., Imran Z., Rafiq M.A., Hasan M.M., Nazir A. Effect of modifiers on structural and optical properties of Titania (TiO_2_) nanoparticles. Proceedings of the IEEE 2011 Saudi International Electronics, Communications and Photonics Conference (SIECPC).

[13] Wypych A., Bobowska I., Tracz M., Opasinska A., Kadlubowski S., Krzywania-Kaliszewska A., Wojciechowski P. (2014). Dielectric properties and characterisation of titanium dioxide obtained by different chemistry methods. J. Nanomater..

[14] Zhou D., Ji Z., Jiang X., Dunphy D.R., Brinker J., Keller A.A. (2013). Influence of material properties on TiO_2_ nanoparticle agglomeration. PLoS ONE.

[15] Zhang L., Zhou Y., Huang M., Sha Y., Tian J., Ye Q. (2014). Effect of nanoparticle surface modification on charge transport characteristics in XLPE/SiO _2_ nanocomposites. IEEE Trans. Dielectr. Electr. Insul..

[16] Abdel-Gawad N.M.K., El Dein A.Z., Mansour D.E.A., Ahmed H.M., Darwish M.M.F., Lehtonen M. (2017). Enhancement of dielectric and mechanical properties of polyvinyl chloride nanocomposites using functionalized TiO_2_ nanoparticles. IEEE Trans. Dielectr. Electr. Insul..

[17] Abdalla M.M., Nasrat L.S., Mansour A.H.I., Othman E.A. (2022). Evaluation of the dielectric strength behavior of rubber blends using feed-forward neural network in different environmental conditions. J. Al-Azhar Univ. Eng. Sect..

[18] Mansour D.A., Abdel-Gawad N.M., El Dein A.Z., Ahmed H.M., Darwish M.M., Lehtonen M. (2020). Recent advances in polymer nanocomposites based on polyethylene and polyvinylchloride for power cables. Materials.

[19] Eldesoky E., Said A., Nawar A., Abdallah M., Kamel S. (2020). High voltage cross-linked polyethylene insulator characteristics improvement using functionalized ZnO nanoparticles. Egypt. J. Chem..

[20] Selvaraj D.E., Vijayaraj R., Sugumaran C.P. (2015). Electrical and Thermal Characterization of Organic Varnish Filled with ZrO_2_ Nano Filler Used in Electrical Machines. J. Electr. Eng. Technol..

[21] Karunarathna P., Chithradewa K., Kumara S., Weerasekara C., Sanarasinghe R., Rathnayake T. Study on dielectric properties of epoxy resin nanocomposites. Proceedings of the IEEE 2019 International Symposium on Advanced Electrical and Communication Technologies (ISAECT).

[22] Lei Z., Men R., Wang F., Li Y., Song J., Shahsavarian T., Fabiani D. (2020). Surface modified nano-SiO_2_ enhances dielectric properties of stator coil insulation for HV motors. IEEE Trans. Dielectr. Electr. Insul..

[23] Bazrgari D., Moztarzadeh F., Sabbagh-Alvani A.A., Rasoulianboroujeni M., Tahriri M., Tayebi L. (2018). Mechanical properties and tribological performance of epoxy/Al_2_O_3_ nanocomposite. Ceram. Int..

[24] Datasheet of Von Roll Isola IMI Varnish 9637 Polyester Varnish for Electrical Insulation. http://www.lookpolymers.com/pdf/Von-Roll-Isola-IMI-Varnish-9637-Polyester-Varnish-for-Electrical-Insulation.pdf.

[25] Zhou Y., Hu J., Dang B., He J. (2017). Effect of different nanoparticles on tuning electrical properties of polypropylene nanocomposites. IEEE Trans. Dielectr. Electr. Insul..

[26] Wang W., Li S. (2019). Improvement of Dielectric Breakdown Performance by Surface Modification in Polyethylene/TiO_2_ Nanocomposites. Materials.

[27] Wang S., Yu S., Li J., Li S. (2020). Effects of functionalized nano-TiO_2_ on the molecular motion in epoxy resin-based nanocomposites. Materials.

[28] Ahmed H.M., Abdel-Gawad N.M., Afifi W.A., Mansour D.E.A., Darwish M.M. Improving Dielectric Properties of Electrical Machines Insulating Varnish Using SiO_2_ Nanoparticles. Proceedings of the 2022 IEEE 23rd International Middle East Power Systems Conference (MEPCON).

[29] Zhao J., Milanova M., Warmoeskerken M.M., Dutschk V. (2012). Surface modification of TiO_2_ nanoparticles with silane coupling agents. Colloids Surf. A Physicochem. Eng. Asp..

[30] Prado L.A.S.A., Sriyai M., Ghislandi M., Barros-Timmons A., Schulte K. (2010). Surface modification of alumina nanoparticles with silane coupling agents. J. Braz. Chem. Soc..

[31] Vyas M.K., Chandra A. (2018). Role of organic/inorganic salts and nanofillers in polymer nanocomposites: Enhanced conduction, rheological, and thermal properties. J. Mater. Sci..

[32] Havriliak S., Negami S. (1966). A complex plane analysis of α-dispersions in some polymer systems. J. Polym. Sci. Part C Polym. Symp..

[33] Karaman H.S., Mansour D.-E.A., Lehtonen M., Darwish M.M.F. (2023). Condition Assessment of Natural Ester–Mineral Oil Mixture Due to Transformer Retrofilling via Sensing Dielectric Properties. Sensors.

[34] (2013). Standard Test Method for Dielectric Breakdown Voltage and Dielectric Strength of Solid Electrical Insulating Materials at Commercial Power Frequencies.

[35] Kim M., Kim S.-H., Lee S.-H. (2020). Finite element analysis of the breakdown prediction for LDPE stressed by various ramp rates of DC voltage based on molecular displacement model. Energies.

[36] Kara A., Kalenderli O., Mardikyan K. (2017). Modeling and analyzing barrier effect on AC breakdown strength of non-uniform air gaps. IEEE Trans. Dielectr. Electr. Insul..

[37] Khan B., Saleem J., Khan F., Faraz G., Rehman N.U. Finite Element Analysis of Electric Field Distribution in the Dielectric Gas R410A as an Alternative to SF_6_ for High Voltage Applications. Proceedings of the IEEE 2018 International Conference on Applied and Engineering Mathematics (ICAEM).

[38] Mahire V.N., Patel V.E., Chaudhari A.B., Gite V.V., Mahulikar P.P. (2016). Silane@ TiO_2_ nanoparticles-driven expeditious synthesis of biologically active benzo [4,5] imidazo [1,2-a] chromeno [4,3-d] pyrimidin-6-one scaffolds: A green approach. J. Chem. Sci..

[39] Wanag A., Sienkiewicz A., Rokicka-Konieczna P., Kusiak-Nejman E., Morawski A.W. (2020). Influence of modification of titanium dioxide by silane coupling agents on the photocatalytic activity and stability. J. Environ. Chem. Eng..

[40] Yamada M., Yoshihara T., Arima H., Kobayashi T. (1997). High–definition image processing system for FE–SEM. Microscopy.

[41] Karaman H.S., El Dein A.Z., Mansour D.-E.A., Lehtonen M., Darwish M.M.F. (2023). Influence of Mineral Oil-Based Nanofluids on the Temperature Distribution and Generated Heat Energy Inside Minimum Oil Circuit Breaker in Making Process. Nanomaterials.

[42] Yang M., Wang Z., Chen S., Xie Q., Mao J., Liu J., Cheng Y. Thermal and electrical properties of BNNPs/TiO_2_-Epoxy three-phase nanocomposites. Proceedings of the IEEE 2017 1st International Conference on Electrical Materials and Power Equipment (ICEMPE).

[43] Wen H., Cheng L., Jiang Y., Zhu T., Chen Z. Comparative study on thermal and electrical properties of EP/SrTiO_3_ and EP/BaTiO_3_ nanocomposites. Proceedings of the 2020 IEEE International Conference on High Voltage Engineering and Application (ICHVE).

[44] Huang C., Qian X., Yang R. (2018). Thermal conductivity of polymers and polymer nanocomposites. Mater. Sci. Eng. R Rep..

[45] Han Z., Fina A. (2011). Thermal conductivity of carbon nanotubes and their polymer nanocomposites: A review. Prog. Polym. Sci..

[46] Quiles-Diaz S., Martínez-Rubí Y., Guan J., Kim K.S., Couillard M., Salavagione H.J., Simard B. (2018). Enhanced thermal conductivity in polymer nanocomposites via covalent functionalization of boron nitride nanotubes with short polyethylene chains for heat-transfer applications. ACS Appl. Nano Mater..

[47] Tessema A., Zhao D., Moll J., Xu S., Yang R., Li C., Kidane A. (2017). Effect of filler loading, geometry, dispersion and temperature on thermal conductivity of polymer nanocomposites. Polym. Test..

[48] Xu Y., Li G. (2009). Strain effect analysis on phonon thermal conductivity of two-dimensional nanocomposites. J. Appl. Phys..

[49] Xu X., Chen J., Zhou J., Li B. (2018). Thermal conductivity of polymers and their nanocomposites. Adv. Mater..

[50] Owolabi A.L., Al-Kayiem H.H., Baheta A.T. (2016). Nanoadditives induced enhancement of the thermal properties of paraffin-based nanocomposites for thermal energy storage. Sol. Energy.

[51] Ebadi-Dehaghani H., Nazempour M. (2012). Thermal conductivity of nanoparticles filled polymers. Smart Nanoparticles Technology.

[52] Zulkifli A. (2012). Polymer dielectric materials. Dielectric Material.

[53] Hosier I.L., Praeger M., Holt A.F., Vaughan A.S., Swingler S.G. (2017). On the effect of functionalizer chain length and water content in polyethylene/silica nanocomposites: Part I—Dielectric properties and breakdown strength. IEEE Trans. Dielectr. Electr. Insul..

[54] Jiang H., Zhang X., Gao J., Guo N. Dielectric Properties of SiO_2_/MMT/LDPE Micro-nano Composites. Proceedings of the 2020 IEEE International Conference on High Voltage Engineering and Application (ICHVE).

[55] Talbi F., David E., Malec D., Mary D. Dielectric Properties of Polyesterimide/SiO_2_ Nanocomposites. Proceedings of the 2019 IEEE Conference on Electrical Insulation and Dielectric Phenomena (CEIDP).

[56] Abdel-Gawad N.M.K., El Dein A.Z., Mansour D.E.A., Ahmed H.M., Darwish M.M., Lehtonen M. (2020). PVC nanocomposites for cable insulation with enhanced dielectric properties, partial discharge resistance and mechanical performance. High Volt..

[57] Ahmed H.M., Hassan M.K., Mauritz K.A., Bunkley S.L., Buchanan R.K., Buchanan J.P. (2014). Dielectric properties of C60 and Sc3N@ C80 fullerenol containing polyurethane nanocomposites. J. Appl. Polym. Sci..

[58] Ahmed H.M., Windham A.D., Al-Ejji M.M., Al-Qahtani N.H., Hassan M.K., Mauritz K.A., Buchanan J.P. (2015). Preparation and preliminary dielectric characterization of structured C60-Thiol-Ene polymer nanocomposites assembled using the Thiol-Ene click reaction. Materials.

[59] Ahmed H.M., Abd El-Fattah Z.M., Anad N.S., Attallah M., El-Bahnasawy H.H. (2023). Thermo-mechanical and opto-electrical study of Cr-doped-ZnO-based polyvinyl chloride nanocomposites. J. Mater. Sci. Mater. Electron..

[60] Li Q., Ju T., Li R., Wang S., Yang Y., Ishida H., Zhu L. (2023). Investigation into the crystal structure–dielectric property correlation in barium titanate nanocrystals of different sizes. Nanoscale.

[61] Kremer F., Schonhals A. (2003). Broadband Dielectric Spectroscopy.

[62] Abdel-Gawad N.M.K., El Dein A.Z., Mansour D.E.A., Ahmed H.M., Darwish M.M.F., Lehtonen M. (2018). Multiple enhancement of PVC cable insulation using functionalized SiO_2_ nanoparticles based nanocomposites. Electr. Power Syst. Res..

[63] Li Y., Kroger M., Liu W.K. (2012). Nanoparticle effect on the dynamics of polymer chains and their entanglement network. Phys. Rev. Lett..

[64] Wong H.C., Cabral J.T. (2010). Nanoparticle aggregation behaviour in polymer nanocomposites: Bulk vs. thin films. J. Phys. Conf. Ser..

[65] Abosheiasha H.F., Mansour D.E.A., Darwish M.A., Assar S.T. (2022). Synthesis and investigation of structural, thermal, magnetic, and dielectric properties of multifunctional epoxy/Li_0.5_Al_0.35_Fe_2.15_O_4_/Al_2_O_3_ nanocomposites. J. Mater. Res. Technol..

[66] Singha S., Thomas M.J. (2008). Dielectric properties of epoxy nanocomposites. IEEE Trans. Dielectr. Electr. Insul..

[67] Preetha P., Thomas M.J. (2011). AC breakdown characteristics of epoxy nanocomposites. IEEE Trans. Dielectr. Electr. Insul..

